# Ketamine may block NMDA receptors in astrocytes causing a rapid antidepressant effect

**DOI:** 10.3389/fnsyn.2012.00008

**Published:** 2012-12-24

**Authors:** Bernhard J. Mitterauer

**Affiliations:** Volitronics-Institute for Basic Research, Psychopathology and Brain Philosophy, University of SalzburgSalzburg, Austria

Depression is associated both with a reduced size of brain regions regulating mood and cognition, including the prefrontal cortex and the hippocampus, and decreased neuronal synapses in these areas. Antidepressants can block or reverse these neuronal deficits although they have limited efficacy and delayed response time of weeks or months. Basic studies show that ketamine, an N-methyl-D-asparate (NMDA) receptor antagonist, produces rapid (within hours) antidepressant effects in patients with chronic depression who are resistant to typical antidepressants (Zarate et al., 2006; Machado-Vieira et al., 2012). The rapid antidepressant actions of ketamine could involve a number of cortical and limbic brain regions, many of which are reported to show alterations of glutamate transmission and synaptic activity (Li et al., 2010). There is experimental evidence that ketamine induces synaptogenesis leading to the synaptogenic hypothesis of depression and treatment response (Duman and Aghajanian, 2012).

Ketamine rapidly reverses the deficit in spine density, as well as the anhedonia and anxiety caused by exposure to chronic stress. However, the mechanisms underlying this action of ketamine, an NMDA antagonist, is as yet not fully understood (Diazgranados et al., 2010). Li et al. (2010) observed that ketamine rapidly activated the mammalian target of rapamacin (mTOR) pathway, leading to increased synaptic signaling proteins and increased number and function of new spine synapses in the prefrontal cortex of rats. Importantly, these researchers admit that the mechanisms underlying rapid antidepressant actions have not been identified so far and that they are likely more complicated than simple NMDA receptor blockade. According to Duman and Aghajanian (2012), the discovery that ketamine rapidly increases the number and function of synaptic connections has focused attention on synaptogenesis as a fundamental process for the treatment of depressive symptoms, and it also suggests that disruption of synaptogenesis and loss of connections underlies the pathophysiology of depression.

In addition, we should also refer to glial-neuronal synaptic units. First of all, perisynaptic astrocytes (glial cells) play a role in promoting the stabilization and maturation of dendritic spines (Stevens, 2008). Life imaging experiments indicate that astrocytes show striking morphological plasticity at the synapse. Time-lapse confocal imaging of dendritic spines and astrocytes in acute slices from transgenic mice revealed that astrocytes rapidly extend and retract filopodia-like processes within a time-scale of minutes (Hirrlinger et al., 2004). Hence, this modulatory function of astrocytes in synaptogenesis has been ignored in the current model of the pathophysiology of depression. There is clear spatiotemporal correlation between the onset of synaptogenesis and the appearance and association of immature astrocytes with neurons at central nervous system synapses (Ullian et al., 2004), suggesting that astrocytes may provide instructive signals to control the formation and development of synapses. Experimental findings indicate that astrocytes have a powerful ability to promote excitatory and inhibitory synaptogenesis (Hu et al., 2007; Stevens, 2008). Moreover, astrocytes exert a modulatory function of neurotransmission in glial-neuronal synaptic units, also called tripartite synapses (Haydon and Carmignoto, 2006). Given the fact that glial-neuronal synaptic units are experimentally well established, we are faced with the question of the role of astrocytes in the pathophysiology of depression. I have hypothesized (Mitterauer, 2009, 2010a,b) that in depression an astrocytic syncytiopathy (glial network disorder)—caused by a downregulation of connexins (proteins of gap junctions)—may lead to an upregulation of astrocytic receptors (Rouach et al., 2002; Suadicani et al., 2003). Such an excess of astrocytic receptors exerts a relative lack of neurotransmitters such that the synaptic information processing is imbalanced. It has been experimentally verified that astrocytes express all receptors for the occupancy with the cognate neurotransmitter types (Hansson and Rönnbäck, 2004). This holds also for NMDA receptors. There is definitive experimental evidence for the existence of functional NMDA receptor expression in human astrocytes (Lee et al., 2010).

Admittedly, ketamine could act on various neuronal and glial cell types and my hypothesis seems to be plausible for signals acting at very low concentrations such as neurotrophins, whereas for glutamate, the main excitatory transmitter of the central nervous system, some supporting arguments are necessary. Principally, upregulation of hippocampal metabotropic glutamate receptor 5 has been identified in temporal lobe epilepsy (Notenboom et al., 2006). Importantly, upregulations of other receptors in astrocytes have also been found in brains with Alzheimer disease (Yu et al., 2012) and with Parkinson disease. The increased expression of protease-activated receptors-1 in astrocytes in substantia nigra pars compacta of Parkinson disease brains is the restorative move taken by the brain to provide neuroprotection against neuronal degeneration (Ishida et al., 2006). Since upregulations of astrocytic receptors occur in brains with neurodegenerative diseases, upregulation of glutamatergic receptors in astrocytes could also be found in brains with major depression. However, the mechanism proposed causing synaptic imbalance is new and must be experimentally verified.

The physiological and pathophysiological considerations shortly discussed allow the following hypothesis: supposing that in therapy-resistant depression a significant excess of NMDA receptors in astrocytes is causing a severe lack of glutamate which cannot be balanced by reuptake inhibitory drugs, the blockade of the excess of NMDA receptors in astrocytes may rapidly balance synaptic information processing. Importantly, since all of the various neurotransmitters and their receptors in the neuronal and glial cell systems may play a role in depression, my model of depression might open a new dimension in the development of antidepressants, especially what the treatment of patients with a chronic depression concerns. It should also be noted that this model is testable by counting or quantifying astrocytic receptors at least in postmortem brains.
